# Successful Management of Heparin-Induced Thrombocytopenia Using Argatroban in a Very Old Woman: A Case Report

**DOI:** 10.1155/2013/586989

**Published:** 2013-03-05

**Authors:** A. Putot, S. Perrin, H. Sordet-Guépet, E. de Maistre, P. Manckoundia

**Affiliations:** ^1^Service de Médecine Interne Gériatrie, Hôpital de Jour, Hôpital de Champmaillot CHU BP 87909, 2 rue Jules Violle, 21079 Dijon Cedex, France; ^2^Service d'Hématologie Biologique Hôpital du Bocage CHU, BP 87909, 21079 Dijon Cedex, France; ^3^INSERM/U1093 Motricité-Plasticité: Performance, Dysfonctionnement, Vieillissement et Technologies d'Optimisation, Université de Bourgogne, Faculté des Sciences du Sport, 21078 Dijon, France

## Abstract

Thrombosis due to heparin-induced thrombocytopenia (HIT) is rare but has a severe prognosis. Its management is not always easy, particularly in old patients with renal insufficiency. A 95-year-old woman was hospitalized for dyspnea. Curative treatment with unfractionated heparin was started because pulmonary embolism was suspected. Disseminated intravascular coagulation was then suspected because of thrombocytopenia, hypoprothrombinemia, hypofibrinogenemia, and a positive ethanol gelation test. The first immunoassay for HIT was negative. On the 12th day of hospitalization, bilateral cyanosis of the toes occurred associated with recent deep bilateral venous and arterial thrombosis at duplex ultrasound. New biological tests confirmed HIT and led us to stop heparin and to start argatroban with a positive clinical and biological evolution. Venous and arterial thrombosis associated with thrombocytopenia during heparin treatment must be considered HIT whatever the biological test results are. Argatroban is a good alternative treatment in the elderly.

## 1. Introduction

Heparin-induced thrombocytopenia (HIT) is an immune-mediated syndrome caused by heparin. Complications range from benign thrombocytopenia to severe thrombosis with risk of death. Its management is not always easy, in particular in elderly patients.

We report a case of severe HIT associated with arterial and venous thrombosis, bilateral distal necrosis of the lower limbs, and disseminated intravascular coagulation (DIC), successfully treated with argatroban. 

## 2. Case Report

A 95-year-old woman was hospitalized for dyspnea and general weakness. Her medical history consisted of heart failure, hypertension, atrial fibrillation, and hemorrhagic stroke. She had been treated with bisoprolol, amiodarone, ramipril, and furosemide.

Clinical examination revealed a cough, dyspnea, irregular heart sounds, bilateral jugular vein dilatation, left basal crepitations, and coldness of the extremities without cyanosis. There was no fever or signs of venous thrombosis, and the peripheral pulses were present.

Initial biological screening showed a platelet count at 153,000 cells/mm^3^, low creatinine clearance at 51 mL/min, high uremia at 13 mmol/L (normal <3.2), and C-reactive protein (CRP) at 94 mg/L (normal <3.2). Arterial blood gas analysis, performed without oxygen, revealed metabolic alkalosis (pH 7.46, normal 7.37–7.43) with hypoxemia at 58 mmHg (normal 76–98), high sodium bicarbonate at 27.3 mmol/L (normal 20–26), and normocapnia at 37 mmHg (normal 35–45). Other parameters, particularly the platelet count, and fibrinogen and coagulation tests, were normal. The electrocardiogram showed atrial fibrillation with known left bundle branch block. The chest X-ray revealed cardiomegaly, interstitial lung disease and right pleural effusion, all of which were known. Transthoracic echocardiography revealed an impaired left ventricular ejection fraction at 45%, moderate mitral insufficiency, and increased right-sided filling pressures without vegetation or intracardiac thrombi. Given the acute right heart failure and high CRP, pulmonary embolism was suspected and curative treatment with unfractionated heparin was started. That diagnosis was not confirmed because the renal insufficiency contraindicated an iodinated lung scan and lung scintigraphy would have been difficult to interpret in view of pulmonary parenchyma deterioration.

**Figure 1:**
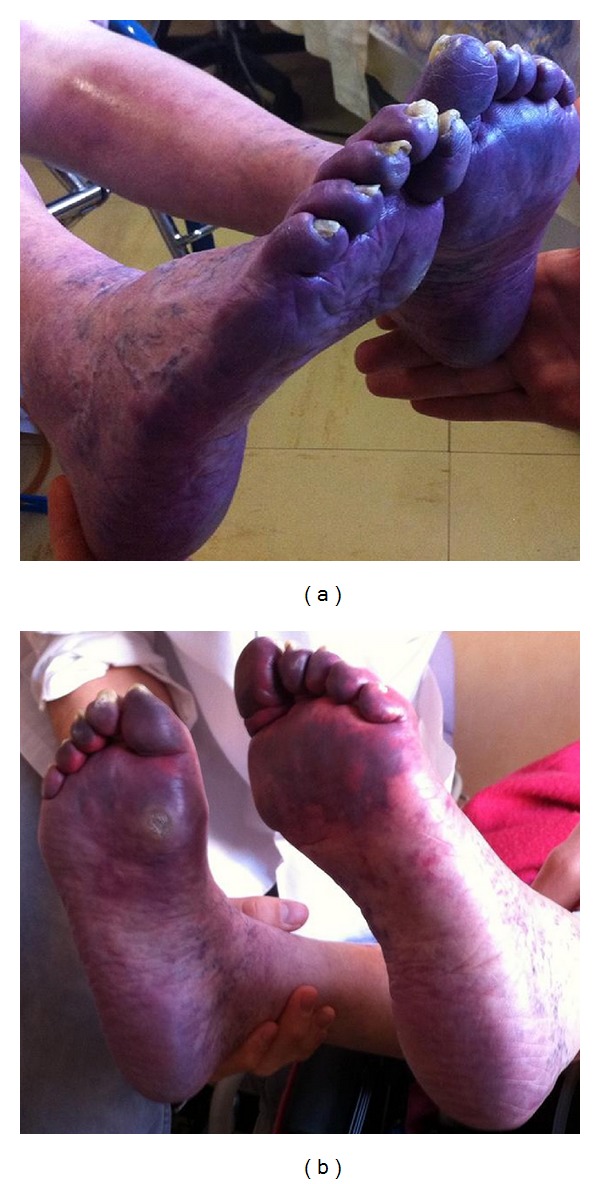
(a) Photograph of the soles on day 13 of hospitalization. (b) Photograph of the soles on day 19 of hospitalization.

On the 6th day of hospitalization, her clinical state was stable and biological screening showed thrombocytopenia at 76,000 cells/mm^3^ and a positive ethanol gelation test. The fibrinogen level decreased to 1.8 g/L three days later (normal 2–4). HIT was suspected but immunoassays (ELISA for anti-PF4/heparin antibodies, rapid particle gel immunoassay PaGIA, Biorad, and a 4T score of 5 at day 6) were negative. DIC was suspected. There was no obvious cause of DIC, that is, no sepsis (blood cultures were negative), no hemodynamic shock, no radiological or clinical argument for a metastatic cancer, no circulating blasts, no acute hepatitis, and no hypo/hyperthermia. On the 12th day of hospitalization, clinical examination showed cyanosis of the toes (Figure 1(a)) and disappearance of the pulse in both feet. Venous and arterial duplex ultrasound revealed recent deep bilateral venous thrombosis in the sural, popliteal, and femoral veins and documented bilateral arterial thrombosis in the posterior and anterior tibial arteries in spite of APTT in the therapeutic range. Plethysmography showed no pulse in the toes, and arterial pressures were near zero. The platelet count was 28,000 cells/mm^3^ and the fibrinogen level was 1.6 g/L. New HIT investigations were performed with positive rapid PaGIA, borderline anti-PF4/heparin ELISA, positive platelet aggregation tests, and a 4T score of 7 (at day 12), indicating a high probability of HIT. Bilateral areas of necrosis appeared quickly on some toes. They were associated with severe pain in the lower limbs. Thus, morphine was prescribed. The unfractionated heparin was stopped and anticoagulation with argatroban was chosen given the exacerbation of the renal insufficiency with creatinine clearance <30 mL/min at the 12th day of hospitalization and the short half-life of the product (less than 1 hour). The low-dose protocol was used (0.5 *μ*g/kg/min), as recommended in intensive care unit. The biological monitoring of argatroban was performed daily with both activated partial thromboplastin time (APTT) (1.5–3 times patient baseline) and a specific coagulation test derived from the thrombin time (thrombin inhibitor Hemoclot, Hyphen Biomed, Neuville sur Oise, France) leading to an estimation of the argatroban concentration. 

A few days after the start of treatment, the pain in the lower limbs vanished, the pulses were restored, cyanosis in the feet diminished (Figure 1(b)), and the platelet count and fibrinogen level both increased two days later. Warfarin was initiated at very low dose (1 mg) after 11 days of argatroban anticoagulation, when the platelet count was over 100,000 cells/mm^3^.

 Despite this clinical and biological improvement, the patient died suddenly during warfarin and argatroban overlap.

## 3. Discussion

Among the known side effects of heparin therapy, thrombocytopenia is the most frequent and dangerous. In 1958, Weismann and Tobin described paradoxical thrombi during heparin therapy [1]. This complication, called type II HIT, is an immune-mediated syndrome characterized by thrombocytopenia, which can be isolated or associated with thrombotic events. Type II HIT is caused by the binding of antibodies (most frequently IgG) to a complex of heparin and platelet factor 4. These antibodies activate platelets through their Fc receptors, causing platelet destruction and the release of prothrombotic platelet-derived microparticles [2].

The incidence of type II HIT varies from 0.5 to 5%, depending on the patient population studied [3]. HIT is far more common in patients treated with unfractionated heparin than in those treated with low-molecular-weight heparin [4]. Fifty percent of patients presenting type II HIT develop venous thrombosis and in 25% of cases pulmonary embolism [5]. Thrombotic events, which concern vessels of all size [6], are most frequently venous [5]. However, arterial thrombosis, especially arterial occlusion of lower legs as in this paper, is possible [6]. These thrombotic events associated with HIT are known as white clot syndrome [7].

In the face of suspected HIT, biological diagnostic tests should be repeated because of the possibility of false negative tests, above all when tests are performed too early (day 6 in our paper), even in a patient who has strong clinical evidence of HIT. Moreover, in case of high clinical suspicion, immunoassays must be associated with platelet aggregation tests. Indeed, the combination of both tests is more reliable than the use of a single test [8]. In our paper, the probability of HIT was high given on the one hand the positivity of new biological tests, including a 4T score of 7, performed when the thrombotic complications occurred, and on the other hand the good response after stopping heparin and beginning argatroban (platelet count, fibrinogen level, thrombotic events). Thus, a pseudo-HIT, which is an alternative cause of thrombocytopenia associated with DIC in patients with extensive thrombosis, could be ruled out.

Disseminated intravascular coagulation (DIC) is observed in some patients with HIT, as in our paper. Biological screening shows relative or absolute hypofibrinogenemia and/or hypoprothrombinemia [9]. Severe limb ischemia due to microvascular or macrovascular thrombosis can occur in these patients [6], even without vitamin K antagonists.

As concerns the management of thrombosis due to HIT, withdrawal of heparin is always necessary and urgent, as is the administration of an alternative antithrombotic agent. In France, three drugs have been approved for anticoagulation in HIT. Among these, two, argatroban and Lepirudin, are direct thrombin inhibitors, and one, Danaparoid, is a heparinoid factor Xa inhibitor [10]. In HIT with thrombosis and renal insufficiency, as described here, the American College of Chest Physicians Evidence-Based Clinical Practice Guidelines recommends the use of argatroban rather than other nonheparin anticoagulants [11]. Indeed, argatroban is metabolized in the liver and is so far the only alternative anticoagulant to heparin that shows pharmacokinetic properties independent of renal function. Argatroban acts fast and has a short elimination half-life estimated at 52 ± 16 min [12], which is particularly interesting in frail patients as in our report. In the case we report, age was not a contraindication for the use of argatroban. Indeed, in a retrospective multicentre database analysis of 118 inpatients (including 62 adults aged ≥65) treated with argatroban for HIT, age was not a significant determinant for argatroban dosage or the thrombotic risk. No patient experienced major bleeding or required amputation, and no patients in the oldest group died or developed new thrombosis [13]. The association of HIT and DIC was another argument to use direct antithrombin agents, as they seem to have a positive impact in this indication, on the basis of small case series [14]. Some authors suggest that argatroban has preventable effect for limb amputation [6]. In this observation, the use of argatroban was relatively easy, with help of biological monitoring that ensure stable blood levels. The use of a specific coagulation test derived from the thrombin time (thrombin inhibitor Hemoclot) with an estimation of argatroban concentration allowed the early detection of overdose with a strong correlation to APTT, despite the absence of a validated therapeutic range. After 11 days of argatroban therapy, biological and clinical improvement allowed antivitamin K therapy relay. Venous limb gangrene and warfarin-induced skin necrosis have been described as complications of HIT during the transition to warfarin therapy [15]. To prevent this, we followed recommendations to ensure adequate levels of anticoagulation and to wait until the platelet count was near normal before introducing small doses of an oral anticoagulant. Unfortunately, though daily biological monitoring (APTT, thrombin inhibitor Hemoclot, International Normalized Ratio) was in the therapeutic zone and though her state improved and became stable, the patient died suddenly.

Age should not be considered a contraindication for argatroban in type II HIT with thrombosis. In our paper, argatroban alleviated lower limb pain due to arterial thrombosis and improved local perfusion; it also allowed quick correction of the thrombocytopenia and hypofibrinogenemia. Argatroban is relatively easy to use in elderly patients because it does not depend on renal function and has a short half-life.

## References

[1] Weismann RE, Tobin RW (1958). Arterial embolism occurring during systemic heparin therapy. *American Medical Association*.

[2] Warkentin TE, Hayward CPM, Boshkov LK (1994). Sera from patients with heparin-induced thrombocytopenia generate platelet-derived microparticles with procoagulant activity: an explanation for the thrombotic complications of heparin-induced thrombocytopenia. *Blood*.

[3] Jang IK, Hursting MJ (2005). When heparins promote thrombosis review of heparin-induced thrombocytopenia. *Circulation*.

[4] Warkentin TE, Levine MN, Hirsh J (1995). Heparin-induced thrombocytopenia in patients treated with low-molecular- weight heparin or unfractionated heparin. *The New England Journal of Medicine*.

[5] Warkentin TE, Kelton JG (1996). A 14-year study of heparin-induced thrombocytopenia. *The American Journal of Medicine*.

[6] Bibbo C, Hatfield PS (2011). Lower extremity manifestations and treatment of heparin-induced thrombocytopenia syndromes: a cohort study. *The Journal of Foot and Ankle Surgery*.

[7] Benhamou AC, Gruel Y, Barsotti J (1985). The white clot syndrome or heparin associated thrombocytopenia and thrombosis (WCS or HATT) (26 cases). *International Angiology*.

[8] Warkentin TE, Greinacher A, Gruel Y, Aster RH, Chong BH (2011). Laboratory testing for heparin-induced thrombocytopenia: a conceptual framework and implications for diagnosis. *Journal of Thrombosis and Haemostasis*.

[9] Warkentin TE, Bernstein RA (2003). Delayed-onset heparin-induced thrombocytopenia and cerebral thrombosis after a single administration of unfractionated heparin. *The New England Journal of Medicine*.

[10] Alatri A, Armstrong AE, Greinacher A (2012). Results of a consensus meeting on the use of argatroban in patients with heparin-induced thrombocytopenia requiring antithrombotic therapy—a European perspective. *Thrombosis Research*.

[11] Linkins LA, Dans AL, Moores LK (2012). Treatment and prevention of heparin-induced thrombocytopenia: antithrombotic therapy and prevention of thrombosis, 9th ed: American College of Chest Physicians Evidence-Based Clinical Practice Guidelines. *Chest*.

[12] Escolar G, Bozzo J, Maragall S (2006). Argatroban: a direct thrombin inhibitor with reliable and predictable anticoagulant actions. *Drugs of Today*.

[13] Bartholomew JR, Pietrangeli CE, Hursting MJ (2007). Argatroban anticoagulation for heparin-induced thrombocytopenia in elderly patients. *Drugs & Aging*.

[14] Bartholomew JR (2005). Transition to an oral anticoagulant in patients with heparin-induced thrombocytopenia. *Chest*.

[15] Srinivasan AF, Rice L, Bartholomew JR (2004). Warfarin-induced skin necrosis and venous limb gangrene in the setting of heparin-induced thrombocytopenia. *Archives of Internal Medicine*.

